# Pain, obesity, adenosine salvage disruption, and smoking behavior mediate the effect of gut microbiota on sleep disorders: results from network Mendelian randomization and 16S rDNA sequencing

**DOI:** 10.3389/fmicb.2024.1413218

**Published:** 2024-07-31

**Authors:** Fu-Jia Li, Ru-Yu Zhang, Jin-Yu Li, Yu-Ning Liu, Zi-Xuan Zhang, Li Du, Yang-Dan-Yu Li, Xu Liu, Wei Zhang, Gui-Yun Cui, Chuan-Ying Xu

**Affiliations:** ^1^Department of Neurology, The Affiliated Hospital of Xuzhou Medical University, Xuzhou, China; ^2^Department of Pulmonary and Critical Care Medicine, First People’s Hospital of Zigong, Zigong, Sichuan, China; ^3^Department of Respiratory and Critical Care Medicine, The Second Affiliated Hospital of Xuzhou Medical University, Xuzhou, China

**Keywords:** Mendelian randomization, triangulating evidence, indirect causality, gut microbiota, sleep disorders

## Abstract

**Objectives:**

The objective of this study is to investigate the indirect causalities between gut microbiota and sleep disorders.

**Methods:**

In stage 1, we utilized 196 gut microbiota as the exposure factor and conducted a two-sample univariable Mendelian randomization (MR) analysis on five sleep disorders: insomnia, excessive daytime sleepiness (EDS), sleep-wake rhythm disorders (SWRD), obstructive sleep apnea (OSA), and isolated REM sleep behavior disorder (iRBD). In stage 2, we validated the MR findings by comparing fecal microbiota abundance between patients and healthy controls through 16S rDNA sequencing. In stage 3, we explored the indirect pathways by which the microbiota affects sleep, using 205 gut microbiota metabolic pathways and 9 common risk factors for sleep disorders as candidate mediators in a network MR analysis.

**Results:**

In stage 1, the univariable MR analysis identified 14 microbiota potentially influencing five different sleep disorders. In stage 2, the results from our observational study validated four of these associations. In stage 3, the network MR analysis revealed that the Negativicutes class and Selenomonadales order might worsen insomnia by increasing pain [mediation: 12.43% (95% CI: 0.47, 24.39%)]. *Oxalobacter* could raise EDS by disrupting adenosine reuptake [25.39% (1.84, 48.95%)]. *Allisonella* may elevate OSA risk via obesity promotion [36.88% (17.23, 56.54%)], while the *Eubacterium xylanophilum* group may lower OSA risk by decreasing smoking behavior [7.70% (0.66, 14.74%)].

**Conclusion:**

Triangulation of evidence from the MR and observational study revealed indirect causal relationships between the microbiota and sleep disorders, offering fresh perspectives on how gut microbiota modulate sleep.

## 1 Introduction

According to the International Classification of Sleep Disorders-Third Edition (ICSD-3), sleep disorders encompass common conditions such as insomnia, excessive daytime sleepiness (EDS), sleep-wake rhythm disorders (SWRD), obstructive sleep apnea (OSA), and isolated rapid eye movement sleep behavior disorder (iRBD) (Sateia, 2014). These disorders contribute to enduring gastrointestinal, psychological, and neurological issues, with certain subtypes recognized as potential precursors to neurodegenerative diseases (Grundgeiger et al., 2014; Dauvilliers et al., 2018; Hyun et al., 2019), posing significant health burdens. Therefore, identifying the risk factors and potential mechanisms of these diseases is crucial for targeted therapy.

Previous studies have illuminated the influential role of the gut microbiota in sleep disorders via the brain-gut axis (Wang et al., 2022). During this process, the microbiome can synthesize specific metabolites, regulate immune and inflammatory responses, and impact the functions of the enteric and vagal nerves to alter sleep dynamics. While the results from these studies have shown significant heterogeneity, we believe that three main factors have limited the robustness of past conclusions. (1) Numerous factors such as diet, medication, smoking, alcohol consumption, chronic diseases, environmental factors, and circadian rhythms can simultaneously influence gut microbiota and sleep (Chang and Kao, 2019; Hasan and Yang, 2019; Sen et al., 2021), leading to substantial confounding bias that is difficult to control. (2) The bidirectional interaction between gut microbiota and sleep complicates the interpretation of correlations found in observational studies (Neroni et al., 2021). (3) The challenges associated with collecting and preserving gut microbiome samples and the high costs of sequencing have resulted in small sample sizes in previous studies, further limiting the replicability of the findings.

Mendelian randomization (MR), which integrates genome-wide association study (GWAS) summary data to form strong instrumental variables for causal inference (Emdin et al., 2017), addresses these limitations effectively. The MR approach relies on GWAS data, which typically encompasses a large sample size. Methodologically, the strength of MR lies in its effective reduction of confounding biases and reverse causation (Sekula et al., 2016). Therefore, we believe that the MR method can effectively overcome the principal limitations of previous studies, making it ideal for exploring relationships between microbiota and disease.

However, previous MR studies examining the relationship between gut microbiota and sleep have several shortcomings: (1) They did not investigate the impact of the microbiota on less common sleep disorders, such as SWRD and iRBD. (2) Previous research lacks non-MR result validation, failing to meet the criteria for triangulation of evidence (Burgess et al., 2019). (3) There was no exploration of how the microbiota influences sleep disorders.

Therefore, in our study, in stage 1, we conducted two-sample univariable MR (UVMR) analyses, to explore the impact of the microbiota on sleep disorders (such as insomnia, EDS, SWRD, OSA, and iRBD). In stage 2, we carried out an observational study to externally validate the UVMR findings. In stage 3, we selected 205 gut microbiota metabolic pathways and 9 common risk factors for sleep disorders as candidate mediators, to investigate the potential mechanisms through which the microbiota indirectly affects sleep.

## 2 Methods

### 2.1 Study design

**Figure 1:**
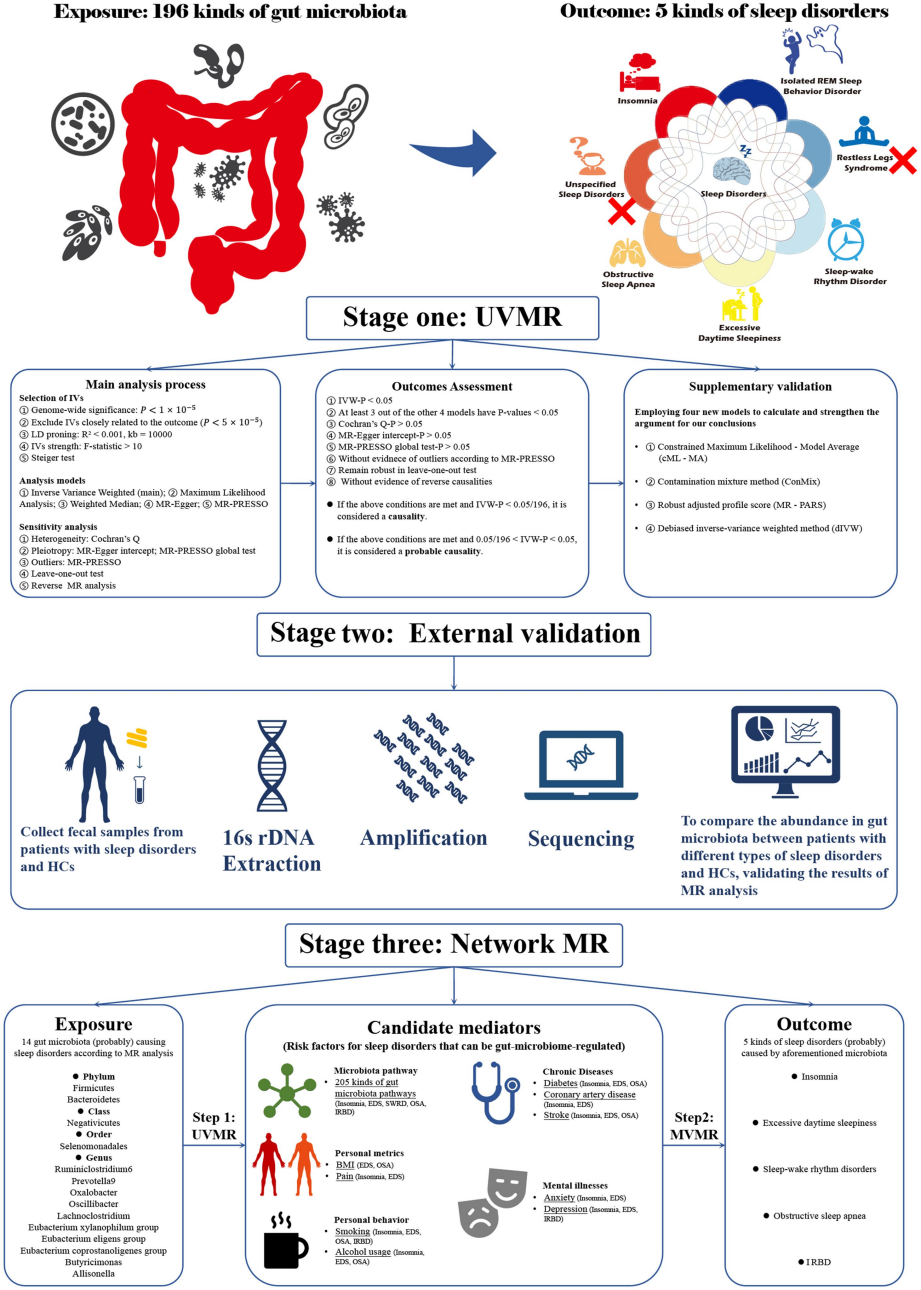
Flowchart of the overall study design. Our research is divided into three stages. Stage 1: Utilizing univariate Mendelian randomization analysis to investigate the causal relationships between gut microbiota and sleep disorders. Stage 2: Employing observational studies as external validation for Mendelian randomization. Stage 3: Using network MR analysis to explore the indirect causal relationship between gut microbiota and sleep disorders. MR, Mendelian randomization; UVMR, univariate MR; MR PRESSO, MR pleiotropy residual sum and outlier; BMI, Body mass index; IVs, Instrumental variables; EDS, excessive daytime sleepiness; SWRD, sleep-wake rhythm disorders; OSA, obstructive sleep apnea; IRBD, isolated REM sleep behavior disorder; HCs, Health controls.

Our study consisted of three stages (Figure 1). In stage 1, we explored the impact of the gut microbiome on sleep disorder incidence using UVMR analysis. In stage 2, we sequenced the 16S rDNA of fecal samples from patients with various sleep disorders and healthy subjects (HCs), comparing microbiome abundance between groups as the external validation for UVMR results. In stage 3, we used network MR to examine potential mediators of microbiome effects on sleep disorders, calculating mediation effects. Using GWAS summary statistics from predominantly European-descendant cohorts, this research adhered to the STROBE-MR guidelines (Skrivankova et al., 2021) (Supplementary Table S1) and the three core assumptions of the MR analysis (Davies et al., 2018), with ethical approval and informed consent detailed in the cited GWAS publications.

### 2.2 Data sources for Mendelian randomization

#### 2.2.1 Exposures

Our research utilized gut microbiome data from the large-scale GWAS meta-analysis by the MiBioGen consortium (Kurilshikov et al., 2021), encompassing 16S rRNA gene sequencing and genotyping data from 18,340 participants across 24 independent cohorts. Given that both the mediators and outcomes in our study pertained to the European population, we selected gut microbiome data exclusively from European descendant from the Integrative Epidemiology Unit (IEU) database (sample size = 14,306, Table 1). In the MiBioGen study, microbiota was categorized into 257 classifications, with 211 suitable for microbial quantitative trait locus (mbQTL) analysis. In our study, we only selected 196 named gut microbiota as the exposure factors, encompassing 9 phyla, 16 classes, 20 orders, 32 families, and 119 genera.

**Table 1:** Data sources in this study.

Phenotype	PMID or GWAS ID	Sample size	Ancestry	Consortium or cohort study
*Exposure*				
Gut microbiome	IEU database ID: ebi-a-GCST90016908 to ebi-a-GCST90017118; PMID: 33462485	14,306	European	MiBioGen consortium
*Outcome*				
Insomnia	IEU database ID: ukb-b-3957	462,341	European	MRC-IEU consortium; UK Biobank
EDS	IEU database ID: ukb-b-5776	460,913	European	MRC-IEU consortium; UK Biobank
SWRD	FinnGen R10 ID: F5_SLEEPWAKE	405,685	European	FinnGen database
OSA	FinnGen R10 ID: G6_SLEEPAPNO	410,385	European	FinnGen database
IRBD	GWAS catalog: GCST90204200; PMID: 36470867	9,447	European The iRBD cohort (*N* cases = 1,061, *N* controls = 8,386) included large cohorts of French, French, Canadian, Italian and British origins, and smaller cohorts from different European populations	International RBD Study Group; French and French-Canadian HYPERGENES Project; Trust Case Control Consortium; Controls from McGill University, LNG, NIA, NIH
*Candidate mediators*				
Gut bacterial pathway (for insomnia, EDS, SWRD, OSA, and iRBD)	IEU database ID: ebi-a-GCST90027446 to ebi-a-GCST90027650; PMID: 35115690	7,738	European (participants from the north of the Netherlands)	Dutch Microbiome Project
Smoking (for OSA and iRBD)	IEU database ID: ieu-b-25 PMID: 30643251	337,334	European	GSCAN; UK Biobank
Smoking (for insomnia and EDS)	IEU database ID: finn-b-SMOKING	138,088	European	FinnGen database
Alcohol usage (for OSA)	IEU database ID: ukb-b-5779	462,346	European	MRC-IEU consortium; UK Biobank
Alcohol usage (for insomnia and EDS)	IEU database ID: finn-b-KRA_PSY_ALCOH	218,792	European	FinnGen database
Body mass index (for EDS and OSA)	IEU database ID: ieu-b-40 PMID: 30124842	681,275	European	GIANT
Pain (for insomnia and EDS)	IEU database ID: finn-b-PAIN	218,369	European	FinnGen database
Type 2 diabetes (for OSA)	IEU database ID: ukb-b-13806	462,933	European	MRC-IEU consortium; UK Biobank
Type 2 diabetes (for insomnia and EDS)	IEU database ID: finn-b-E4_DM2	215,654	European	FinnGen database
Coronary artery disease (for insomnia and EDS)	IEU database ID: finn-b-I9_CHD	218,792	European	FinnGen database
Stroke (for OSA)	IEU database ID: ukb-b-6358	462,933	European	MRC-IEU consortium; UK Biobank
Stroke (for insomnia and EDS)	IEU database ID: finn-b-C_STROKE	180,862	European	FinnGen database
Anxiety (for insomnia and EDS)	IEU database ID: finn-b-F5_ALLANXIOUS	210,623	European	FinnGen database
Depression (for insomnia, EDS, and iRBD)	IEU database ID: finn-b-F5_DEPRESSIO	215,644	European	FinnGen database

MRC-IEU, Medical Research Council Integrative Epidemiology Unit; LNG, Laboratory of Neurogenetics; NIA, National Institute on Aging; NIH, National Institutes of Health; DBDS, Danish Blood Donor Study; GSCAN, Sequencing Consortium of Alcohol and Nicotine use; GIANT, Genetic Investigation of ANthropometric Traits.

#### 2.2.2 Candidate mediators

To explore the potential indirect causalities, we endeavored to identify probable mediators lying in the pathway between gut microbiota and sleep disorders. Candidate mediators must meet two criteria: (1) they are recognized as risk factors for sleep disorders, and (2) they can be regulated by gut microbiota. Through a comprehensive review of the literature (Supplementary Table S11), we identified 205 gut microbiota pathways as common mediators across all “microbiota-sleep disorder” relationships. For the “microbiota-insomnia” relationship, candidate mediators include smoking, alcohol consumption, pain, anxiety, depression, diabetes, coronary heart disease, and stroke. In the “microbiota-EDS” relationship, candidate mediators include BMI, smoking, alcohol consumption, pain, anxiety, depression, diabetes, coronary heart disease, and stroke. For the “microbiota-OSA” relationship, mediators include BMI, smoking, alcohol consumption, diabetes, and stroke. In the “microbiota-iRBD” relationship, smoking and depression were identified as candidate mediators.

The sources for all GWAS data on these mediators are presented in Table 1. To minimize interference from sample overlap, we selected mediator data from datasets independent of exposure and outcome factors.

#### 2.2.3 Outcomes

According to ICSD-3, we incorporated GWAS data for five prevalent sleep disorders, namely, insomnia, EDS, SWRD, OSA, and iRBD as outcomes (Sateia, 2014). The UK Biobank^1^ provided GWAS data for insomnia [International Classification of Diseases version-10 (ICD10) codes G47.0, F51.0; data collected from 2012 to 2018] and EDS (ICD10 codes G47.1, F51.1; data collected from 2012 to 2018), which included 462,341 and 460,913 samples, respectively.

The data for SWRD (ICD10 codes G47.2, F51.2; data collected from 2017 to 2023) and OSA (ICD10 codes G37.3; data collected from 2017 to 2023) were sourced from the Finnish Biobank R10,^2^ containing 405,685 and 410,385 samples, respectively.

In 2022, Krohn et al. (2022) contributed to the largest iRBD GWAS dataset, comprising 9,447 samples (including 1,061 cases from the International RBD study group and 8,386 controls), and iRBD cases were diagnosed following the International Classification of Sleep Disorders (2nd or 3rd Edition), including video polysomnography assessments.

All these datasets were selected for their public availability and large sample sizes to ensure sufficient statistical power for causality detection and mitigate potential biases arising from small sample sizes (Thompson et al., 2016).

### 2.3 Stage 1: univariable Mendelian randomization

#### 2.3.1 Selection of instrumental variables

Given the exceedingly small number of eligible instrumental variables (IVs) below the genome-wide significance threshold (*p* < 5 × 10^−8^), we opted for a relatively less stringent threshold (*p* < 5 × 10^−5^), as informed by previous studies (Li et al., 2022; Liu et al., 2022; Cui et al., 2023). The instrument strength was estimated using the F-statistic (Huang et al., 2021), and an *F*-statistic exceeding 10 suggests the absence of weak IV bias (Burgess and Thompson, 2011). We selected SNPs with a minor allele frequency (MAF) ≥0.01. We clumped independent SNPs based on European ancestry reference data (1,000 Genomes Project, *r*^2^ > 0.001, genomic region = 10,000 kb). Summary statistics were harmonized on alleles positively associated with exposures. SNPs that were ambiguous palindromes (A/T, C/G) and had an MAF greater than 0.42 were excluded (Jiang et al., 2023). Furthermore, to mitigate reverse causation bias, we excluded SNPs that were significantly associated with the outcome (*p* < 5 × 10^−5^) (Park, 2023) and utilized the Steiger method for further filtering (Hemani et al., 2017).

#### 2.3.2 Primary and secondary analyses of UVMR

The inverse-variance weighted (IVW) method (Burgess et al., 2017) served as our primary analysis esteemed for its accuracy and power in estimating causal effects when all selected SNPs are valid IVs (Burgess et al., 2013). To assess the robustness of the IVW results under various assumptions, we used additional MR methods robust to pleiotropy as secondary analyses. These included the maximum likelihood (ML) method (Xue et al., 2021), the weighted median (WM) (Bowden et al., 2016) approach, MR-Egger regression (Bowden et al., 2015), and the Mendelian randomization pleiotropy residual sum and outlier (MR-PRESSO) test (Verbanck et al., 2018), offering a comprehensive evaluation of the causal inferences drawn.

#### 2.3.3 Sensitivity analyses of UVMR

We used several sensitivity analysis methods. First, one primary analysis method and four secondary analysis methods were used to assess the robustness of the results. Second, Cochran’s *Q* statistic assessed heterogeneity (indicative of potential pleiotropy) in IVW estimates. Third, horizontal pleiotropy was evaluated using the *p*-value for the intercept in MR-Egger (Bowden et al., 2015) and the *p*-value for the global test in MR-PRESSO analysis (Verbanck et al., 2018). Fourth, outlier SNPs identified by MR-PRESSO were excluded, with the remaining SNPs subjected to repeated analysis. Fifth, the leave-one-out method tested whether the MR analysis results were driven by any single SNP. Finally, reverse MR analysis examined the potential for reverse causation to affect the outcomes.

For robust conclusions, we applied stringent criteria to positive results. A finding was considered “probable causality” only if the *p*-value from the IVW estimate was <0.05, at least three of the four secondary analytical methods supported the IVW results (*p* < 0.05), with no evidence of influence on heterogeneity, horizontal pleiotropy, outliers, or reverse causation. Furthermore, a result was deemed “causality” only if the *p*-value from the IVW estimate was <0.05/196 (passing the Bonferroni correction).

#### 2.3.4 Supplementary validations for UVMR results

Moreover, various novel MR models have been developed in recent years such as (1) the constrained maximum likelihood-model average method (cML-MA), eliminating biases caused by correlated and uncorrelated pleiotropy (Xue et al., 2021); (2) the contamination mixture method (ConMix), estimating the size of causal effects in the presence of invalid instruments with the lowest mean squared error in a range of realistic scenarios (Burgess et al., 2020); (3) the robust adjusted profile score (MR-RAPS), enabling robust causal inference under weak instrument variable conditions (Yu et al., 2023); (4) the debiased inverse-variance weighted method (dIVW), further mitigating bias from weak instrument variables in IVW analysis. We selected these four new algorithms as supplementary validations to check the robustness of results classified as “probable causality” or “causality.”

### 2.4 Stage 2: external validations

#### 2.4.1 Observational study design

The UVMR analysis revealed that 14 gut microbiota might influence the risk of 5 categories of sleep disorders. To further corroborate the findings, we collected fecal samples from patients with these sleep disorders and HCs. We then performed 16S rDNA sequencing to compare the differences in microbiome proportions between groups, checking for consistency with the trends predicted by the UVMR analysis.

#### 2.4.2 Study subjects

All patients were recruited from the Department of Neurology at the Affiliated Hospital of Xuzhou Medical University. HCs were either hospitalized for health check-ups or caregivers of patients. Participants were excluded if they had recently taken probiotics, were on long-term medication, had severe neurological, mental, or systemic diseases, or had familial genetic disorders.

In this study, 16 insomnia patients, 16 EDS patients, 14 SWRD patients, 10 OSA patients, and 16 pRBD (probable REM Sleep Behavior Disorder) patients were recruited. To diagnose insomnia, we applied the Insomnia Severity Index (Bastien et al., 2001), considering patients with scores above 10 as having insomnia. This threshold demonstrated a sensitivity of 86.1% and a specificity of 87.7% (Morin et al., 2011). The Epworth Sleepiness Scale was used to diagnose excessive daytime sleepiness, with scores over 10 indicating the condition, which showcases a sensitivity of 93.5% and a specificity of 100% (Johns, 2000). We used the RBD-HK scale, developed by Li et al. (2010), to assess patients for symptoms of RBD. In this scale, individuals scoring above 17 were classified as probable RBD (pRBD) patients. At this threshold of 17 points, the scale demonstrated a sensitivity of 85% and a specificity of 81% (Shen et al., 2014). Additionally, we diagnosed obstructive sleep apnea (OSA) patients following the AASM-2012 diagnostic criteria, while sleep-wake rhythm disorder diagnoses adhered to the ICSD-3 criteria. The HCs did not exhibit any type of sleep disorder.

Data on characteristics such as sex, age, BMI, and years of education were collected for each participant. To improve comparability between groups, we included patients whose baseline characteristics were similar to those of HCs. Written informed consent was collected from all participants prior to the commencement of the study. This research was approved by the Ethics Committee of the Affiliated Hospital of Xuzhou Medical University (No. XYFY2022-KL262-01, No. XYFY2023-KL266-01).

#### 2.4.3 Laboratory assessment and preprocessing of raw data

Three fecal samples were obtained from each participant during their first bowel movement. To minimize environmental contamination, samples were taken from the central portion of the stool and placed into sterile containers. These samples were then transported on ice, stored at −80°C, and, subsequently, underwent DNA isolation. After completing the collection of all samples, 16S rDNA sequencing was performed. Total DNA was extracted from the fecal samples using the PF Mag-Bind Stool DNA Kit (Omega Bio-Tek, United States). An optimized primer set (forward primer 338F: ACTCCTACGGGAGGCAGCAG, reverse primer 806R: GGACTACHVGGGTWTCTAAT; Sangon Biotech, Shanghai, China) was used to amplify the multiplex primer library covering the V3–V4 region of the 16S rDNA gene (Liu et al., 2016). Sequencing was conducted on the Illumina PE300/PE250 sequencer (Illumina, United States) by Shanghai Megi Biomedical Technology Co., Ltd. Raw sequence data were quality-controlled using Fastp software (Chen et al., 2018) (https://github.com/OpenGene/fastp, version 0.20.0) and assembled using FLASH software (Magoč and Salzberg, 2011) (http://www.cbcb.umd.edu/software/flash, version 1.2.11). Sequences were clustered into OTUs based on 97% similarity using UPARSE software (Edgar, 2013) (http://drive5.com/uparse/, version 11). Mitochondrial sequences annotated in all samples were removed. All sample sequences were rarefied to 20,000, and after rarefaction, the average sequence coverage (Good’s coverage) for each sample remained at 99.09%. OTU taxonomic annotation was performed by aligning against the Silva 16S rRNA gene database (v138) using the RDP classifier (Wang et al., 2007) (http://rdp.cme.msu.edu/, version 2.13) with a confidence threshold of 70%. Community composition for each sample was then determined at various taxonomic levels.

#### 2.4.4 Statistical analyses

We initially compared baseline characteristics between participants with various sleep disorders and those in the control group. Continuous variables, presented as means and standard deviations, were analyzed using an independent-sample *t*-test. Categorical variables were expressed as proportions and assessed using the chi-square test. All aforementioned analyses were conducted using R version 4.3.1.

All microbiota-related data analyses were conducted on the Majorbio Cloud Platform.^3^ Specifically, the alpha diversity, ACE, Shannon, and Simpson indices were calculated using mothur software (Schloss et al., 2009) (http://www.mothur.org/wiki/Calculators, version v1.30.2), with group differences in alpha diversity assessed via the Wilcoxon test.

For the gut microbiota showing “probable causalities” or “causalities” with sleep disorders in stage 1 through UVMR analysis, we compared their proportions between the patients and HC groups using the Wilcoxon test and examined whether the direction of differences aligns with the trend observed in the MR analysis.

### 2.5 Stage 3: network Mendelian randomization

We utilized the UVMR method to estimate the overall effect of gut microbiota on sleep disorders. Furthermore, we used network MR (or two-step MR) analysis to explore the pathways through which gut microbiota may influence sleep disorders. Stage 1 involved using UVMR to estimate the causal effect of genetically determined gut microbiota on each candidate mediator (β1). Stage 2 utilized MVMR to estimate the causal effect of each candidate mediator on sleep disorders, adjusting for microbiome abundance (β2).

We proceeded to evaluate the magnitude of the mediating effect using the product of coefficients method (β1 × β2) when candidate mediators met the following three criteria: (1) UVMR analysis demonstrating the capacity of the microbiota to influence the mediator; (2) MVMR analysis confirming that, after adjusting for microbiota variations, the mediator independently affects the incidence of sleep disorders; and (3) the direction of the mediating effect must be consistent with the direction of the overall effect.

The proportion of the mediating effect was calculated by dividing the mediating effect by the overall effect. The standard error of the mediating effect was derived using the delta method (Carter et al., 2019).

In step 1, we conducted heterogeneity and horizontal pleiotropy analyses as part of the sensitivity analysis for UVMR. In step 2, we applied the MVMR-Egger method to confirm the robustness of our MVMR-IVW findings.

All MR analyses were conducted using the TwoSampleMR (0.5.7) and MR-PRESSO packages in R, version 4.3.1.

## 3 Results

### 3.1 Stage 1: causalities between gut microbiota and sleep disorders through UVMR analysis

Our study identified 14 potential causal relationships: 14 microbiota may influence the risk of 5 types of sleep disorders. Among them, 10 causal relationships were classified as “probable causalities,” while 4 were classified as “causalities” (Table 2).

**Table 2:** Exploring the causal relationships between gut microbiota and sleep disorders with the UVMR method.

Exposure	Outcome	Method	*n*SNP	*p*-value	β (95% CI)	Judgment	Supplementary validations
Phylum Firmicutes	Insomnia	IVW	17	1.50 × 10^−2^	0.017 (0.003, 0.032)	Probable causality	cML-MA (+)
		ML		5.99 × 10^−3^	0.018 (0.005, 0.031)		ConMix (+)
		MR Egger		3.18 × 10^−1^	0.017 (−0.015, 0.048)		dIVW (+)
		MR PRESSO		2.71 × 10^−2^	0.017 (0.003, 0.032)		MR-PAPS (+)
		WM		2.30 × 10^−3^	0.027 (0.010, 0.045)		
Class Negativicutes	Insomnia	IVW	12	1.08 × 10^−4^	0.032 (0.016, 0.049)	**Causality**	cML-MA (+)
		ML		1.60 × 10^−4^	0.033 (0.016, 0.050)		ConMix (+)
		MR Egger		1.47 × 10^−1^	0.041 (−0.010, 0.093)		dIVW (+)
		MR PRESSO		8.80 × 10^−4^	0.032 (0.018, 0.047)		MR-PAPS (+)
		WM		1.00 × 10^−4^	0.045 (0.022, 0.067)		
Order Selenomonadales	Insomnia	IVW	12	1.08 × 10^−4^	0.032 (0.016, 0.049)	**Causality**	cML-MA (+)
		ML		1.60 × 10^−4^	0.033 (0.016, 0.050)		ConMix (+)
		MR Egger		1.47 × 10^−1^	0.041 (−0.010, 0.093)		dIVW (+)
		MR PRESSO		8.80 × 10^−4^	0.032 (0.018, 0.047)		MR-PAPS (+)
		WM		1.25 × 10^−4^	0.045 (0.022, 0.068)		
Genus *Lachnoclostridium*	Insomnia	IVW	12	8.05 × 10^−4^	0.035 (0.015, 0.056)	Probable causality	cML-MA (+)
		ML		6.01 × 10^−5^	0.038 (0.019, 0.056)		ConMix (+)
		MR Egger		7.70 × 10^−1^	0.011 (−0.062, 0.084)		dIVW (+)
		MR PRESSO		6.47 × 10^−3^	0.035 (0.015, 0.056)		MR-PAPS (+)
		WM		3.49 × 10^−2^	0.028 (0.002, 0.055)		
Phylum Bacteroidetes	EDS	IVW	12	1.51 × 10^−2^	0.013 (0.003, 0.024)	Probable causality	cML-MA (−)
		ML		1.68 × 10^−2^	0.014 (0.002, 0.025)		ConMix (+)
		MR Egger		6.47 × 10^−1^	0.005 (−0.017, 0.028)		dIVW (+)
		MR PRESSO		3.30 × 10^−2^	0.013 (0.003, 0.024)		MR-PAPS (+)
		WM		2.97 × 10^−2^	0.017 (0.002, 0.032)		
Genus *Butyricimonas*	EDS	IVW	13	1.20 × 10^−3^	0.014 (0.006, 0.023)	Probable causality	cML-MA (+)
		ML		1.39 × 10^−3^	0.015 (0.006, 0.024)		ConMix (+)
		MR Egger		7.50 × 10^−1^	0.005 (−0.024, 0.034)		dIVW (+)
		MR PRESSO		5.18 × 10^−3^	0.014 (0.006, 0.022)		MR-PAPS (+)
		WM		6.93 × 10^−3^	0.016 (0.004, 0.028)		
Genus *Eubacterium eligens* group	EDS	IVW	8	2.50 × 10^−2^	0.015 (0.002, 0.028)	Probable causality	cML-MA (+)
		ML		2.59 × 10^−2^	0.015 (0.002, 0.028)		ConMix (+)
		MR Egger		1.35 × 10^−1^	0.048 (−0.006, 0.103)		dIVW (+)
		MR PRESSO		1.92 × 10^−2^	0.015 (0.005, 0.024)		MR-PAPS (+)
		WM		3.05 × 10^−2^	0.019 (0.002, 0.035)		
Genus *Oxalobacter*	EDS	IVW	10	3.45 × 10^−5^	0.014 (0.007, 0.020)	**Causality**	cML-MA (+)
		ML		5.20 × 10^−5^	0.014 (0.007, 0.021)		ConMix (+)
		MR Egger		4.82 × 10^−1^	0.012 (−0.020, 0.045)		dIVW (+)
		MR PRESSO		2.52 × 10^−3^	0.014 (0.007, 0.020)		MR-PAPS (+)
		WM		4.41 × 10^−4^	0.016 (0.007, 0.024)		
Genus *Prevotella9*	SWRD	IVW	15	3.05 × 10^−2^	0.631 (0.059, 1.202)	Probable causality	cML-MA (−)
		ML		6.90 × 10^−3^	0.650 (0.178, 1.122)		ConMix (+)
		MR Egger		6.83 × 10^−2^	1.602 (0.023, 3.182)		dIVW (+)
		MR PRESSO		4.83 × 10^−2^	0.631 (0.059, 1.202)		MR-PAPS (+)
		WM		3.43 × 10^−2^	0.742 (0.055, 1.429)		
Genus *Ruminiclostridium6*	SWRD	IVW	16	5.38 × 10^−3^	−0.819 (−1.396, −0.242)	Probable causality	cML-MA (+)
		ML		7.32 × 10^−3^	−0.798 (−1.382, −0.215)		ConMix (+)
		MR Egger		2.17 × 10^−2^	−1.925 (−3.385, −0.465)		dIVW (+)
		MR PRESSO		1.39 × 10^−2^	−0.819 (−1.396, −0.242)		MR-PAPS (+)
		WM		1.88 × 10^−3^	−1.218 (−1.986, −0.450)		
Genus *Allisonella*	OSA	IVW	8	1.05 × 10^−3^	0.070 (0.028, 0.112)	Probable causality	cML-MA (+)
		ML		1.35 × 10^−3^	0.071 (0.028, 0.115)		ConMix (+)
		MR Egger		7.70 × 10^−1^	0.044 (−0.239, 0.328)		dIVW (+)
		MR PRESSO		1.46 × 10^−3^	0.070 (0.043, 0.097)		MR-PAPS (+)
		WM		7.25 × 10^−3^	0.070 (0.019, 0.121)		
Genus *Eubacterium xylanophilum* group	OSA	IVW	9	1.31 × 10^−4^	−0.146 (−0.221, −0.071)	**Causality**	cML-MA (+)
		ML		2.75 × 10^−4^	−0.145 (−0.222, −0.067)		ConMix (+)
		MR Egger		3.07 × 10^−2^	−0.308 (−0.532, −0.084)		dIVW (+)
		MR PRESSO		1.00 × 10^−3^	−0.146 (−0.203, −0.089)		MR-PAPS (+)
		WM		5.47 × 10^−4^	−0.174 (−0.273, −0.075)		
Genus *Eubacterium coprostanoligenes* group	iRBD	IVW	13	1.42 × 10^−2^	0.681 (0.137, 1.224)	Probable causality	cML-MA (+)
		ML		9.18 × 10^−3^	0.714 (0.177, 1.252)		ConMix (−)
		MR Egger		5.95 × 10^−1^	−0.605 (−2.771, 1.562)		dIVW (+)
		MR PRESSO		3.04 × 10^−2^	0.681 (0.137, 1.224)		MR-PAPS (+)
		WM		2.80 × 10^−2^	0.837 (0.090, 1.584)		
Genus *Oscillibacter*	iRBD	IVW	13	7.97 × 10^−3^	0.504 (0.132, 0.876)	Probable causality	cML-MA (+)
		ML		6.46 × 10^−3^	0.531 (0.149, 0.913)		ConMix (+)
		MR Egger		4.91 × 10^−1^	0.584 (−1.025, 2.193)		dIVW (+)
		MR PRESSO		7.65 × 10^−3^	0.504 (0.195, 0.812)		MR-PAPS (+)
		WM		2.31 × 10^−2^	0.574 (0.079, 1.070)		

*n*SNPs, the number of single nucleotide polymorphisms (SNPs); IVW, inverse variance weighted; ML, maximum likelihood; WM, weighted median; MR PRESSO, Mendelian randomization pleiotropy residual sum and outlier; cML-MA, constrained maximum likelihood; ConMix, contamination mixture method; dIVW, debiased inverse-variance weighted method; MR-PAPS, robust adjusted profile score. In the ‘Judgment’ column, if the result is determined as ‘causality,’ the text should be bolded.

Our analysis identified four microbiota associated with an increased risk of insomnia. Specifically, the class Negativicutes [Beta (95% confidence interval) = 0.035 (0.015, 0.056), *p* = 1.08 × 10^−4^, IVW] and the order Selenomonadales [Beta = 0.032 (0.016, 0.049), *p* = 1.08 × 10^−4^, IVW] were found to have a “causality” with insomnia. The phylum Firmicutes [Beta = 0.017 (0.003, 0.032), *p* = 1.50 × 10^−2^, IVW] and the genus *Lachnoclostridium* [Beta = 0.035 (0.015, 0.056), *p* = 8.05 × 10^−4^, IVW] showed “probable causalities” with the outcome. Secondary analyses using ML, WM, and MR-PRESSO models supported these findings. Supplementary validations with the cML-MA, ConMix, dIVW, and MR-PAPS models also supported the IVW conclusions (Supplementary Tables S3, S6).

Four microbiota were linked to an increased risk of EDS. The genus *Oxalobacter* [Beta = 0.014 (0.007, 0.020), *p* = 3.45 × 10^−5^, IVW] showed a “causality” with EDS. The phylum Bacteroidetes [Beta = 0.013 (0.003, 0.024), *p* = 1.51 × 10^−2^, IVW], genus *Butyricimonas* [Beta = 0.014 (0.006, 0.023), *p* = 1.20 × 10^−3^, IVW], and genus *Eubacterium eligens* group [Beta = 0.015 (0.002, 0.028), *p* = 2.50 × 10^−2^, IVW] exhibited “probable causalities” with EDS. These associations were supported by secondary analyses (ML, WM, and MR-PRESSO) and supplementary validations with all four new models, except for the cML-MA method, which did not replicate the causality between the phylum Bacteroidetes and EDS.

Two microbiota were associated with SWRD, both classified as “probable causalities.” The genus *Prevotella9* [Beta = 0.631 (0.059, 1.202), *p* = 3.05 × 10^−2^, IVW] increased SWRD risk, while the genus *Ruminiclostridium6* [Beta = −0.819 (−1.396, −0.242), *p* = 5.38 × 10^−3^, IVW] had a protective effect. These findings were supported by secondary analyses (ML, WM, and MR-PRESSO) and all methods for supplementary validations, except for the cML-MA method, which did not replicate the causality between the genus *Prevotella9* and SWRD.

Two microbiota were found to affect the incidence of OSA. The genus *Allisonella* [Beta = 0.070 (0.028, 0.112), *p* = 1.05 × 10^−3^, IVW] was associated with an increased risk of OSA, a finding classified as “probable causality.” The genus *Eubacterium xylanophilum* group [Beta = −0.146 (−0.221, −0.071), *p* = 1.31 × 10^−4^, IVW] was found to have a protective effect on OSA, with evidence level “causality.” These results were supported by secondary analyses from three methods and supplementary validations with all four new models.

Finally, two microbiota were linked to an increased risk of iRBD, both classified as “probable causalities”: the genus *Eubacterium coprostanoligenes* group [Beta = 0.681 (0.137, 1.224), *p* = 1.42 × 10^−2^, IVW] and the genus *Oscillibacter* [Beta = 0.54 (0.132, 0.876), *p* = 7.97 × 10^−3^, IVW]. These associations were supported by secondary analyses (ML, WM, and MR-PRESSO) and supplementary validations, except for the ConMix method, which did not replicate the causality between the genus *Eubacterium coprostanoligenes* group and iRBD.

The results of sensitivity analyses suggest that all findings remain unaffected by weak instrument variables, heterogeneity, horizontal pleiotropy, outliers, and reverse causations (Supplementary Tables S2, S4, S5, S7, S8).

### 3.2 Stage 2: external validation of stage 1 results from an observational study

**Figure 2:**
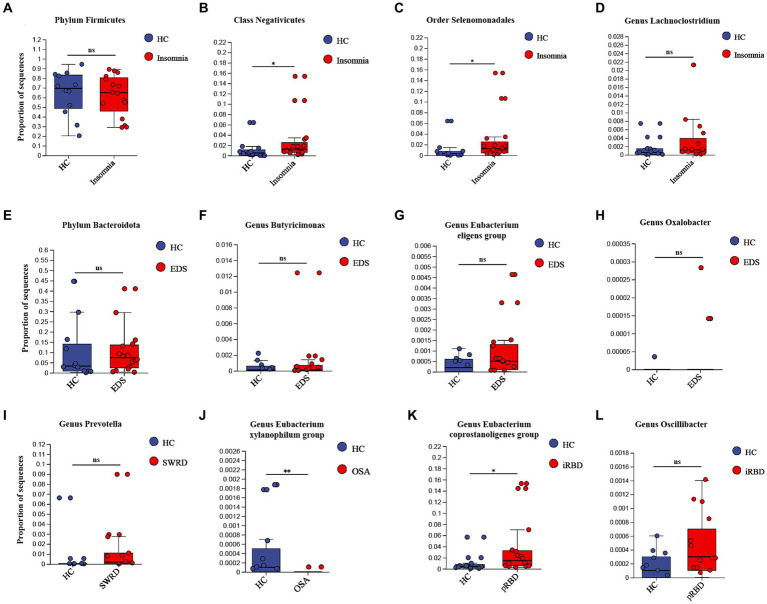
External validation of UVMR results. For those identified “causalities” and “probable causalities” from stage 1, we compared the differences in gut microbiota between different sleep disorder patients and healthy controls using 16S rDNA sequencing to determine if they exhibit a consistent trend with the results of UVMR. pRBD, patients with probable REM sleep behavior disorders. Statistical significant differences are annotated with “*,” while non-significant differences are annotated with “ns”.

Patients with various sleep disorders and HCs showed no significant differences in gender, age, BMI, or years of education (Supplementary Table S9). Compared to HCs, patients with insomnia, SWRD, and OSA exhibited significantly lower alpha diversity. For the 14 types of microbiota identified by UVMR as potentially having causal relationships with sleep disorders, we assessed differences in their proportions between groups using the Wilcoxon test (Supplementary Table S10). We found that the proportion of the class Negativicutes and the order Selenomonadales was significantly higher in the insomnia group compared to HCs (Figures 2B,C); the proportion of the genus *Eubacterium xylanophilum* group was significantly lower in the OSA group than in HCs (Figure 2J); and the proportion of the genus *Eubacterium coprostanoligenes* group was significantly higher in the pRBD group compared to HCs (Figure 2K). No significant differences were found in the remaining 10 types of microbiota between groups (Figure 2).

### 3.3 Stage 3: indirect causalities between gut microbiota and sleep disorders from the network MR analysis

**Figure 3:**
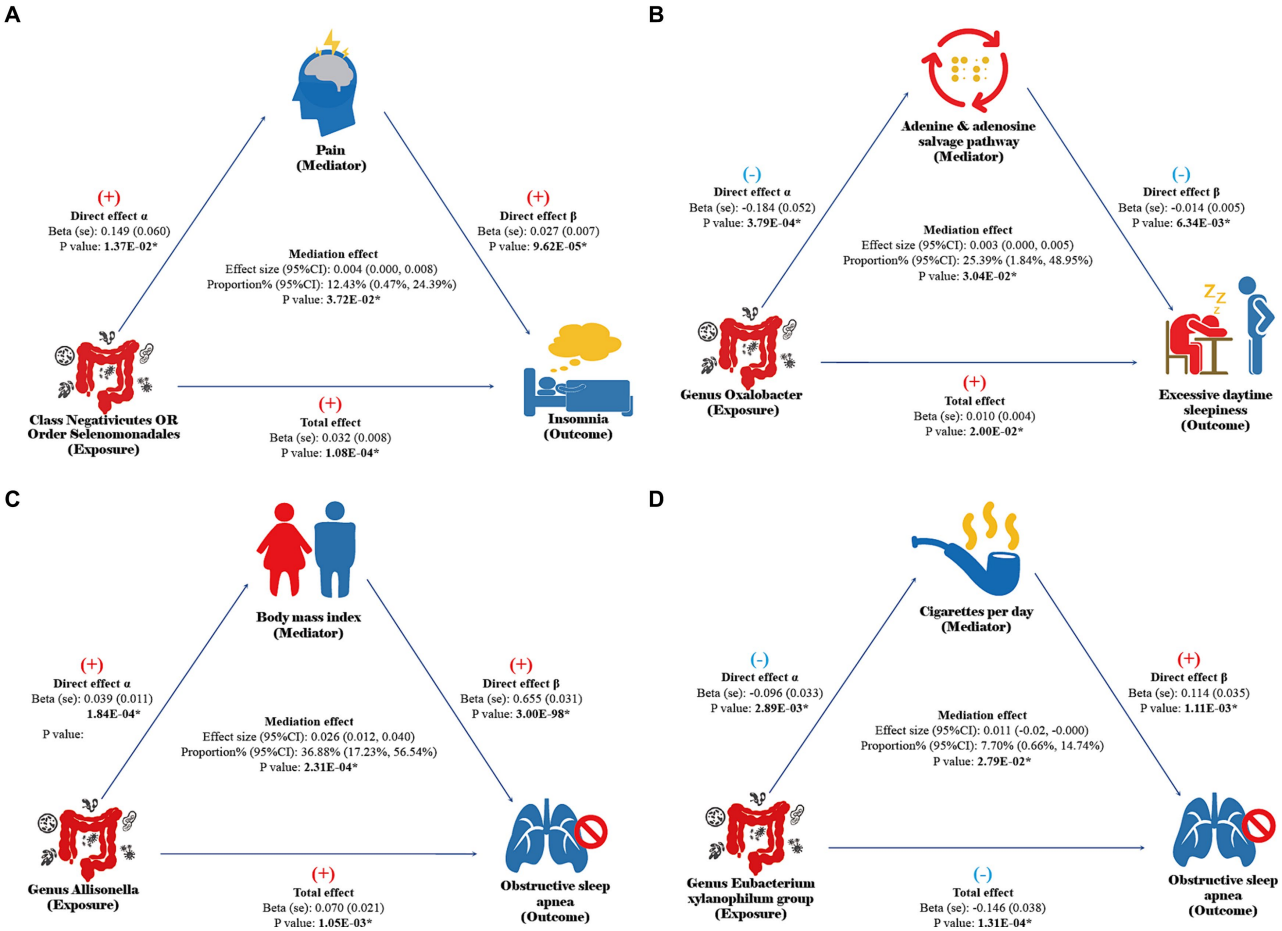
Results of network MR. Direct effect α: the causal effect of gut microbiota on mediators in UVMR analysis. Direct effect β: the causal effect of mediators on sleep disorders after adjusting for gut microbiota abundance in MVMR analysis. Total effect: the total effect of gut microbiota on sleep disorders in UVMR analysis. Mediation effect: the indirect effect of gut microbiota on sleep disorders through mediators calculated by the coefficient product method. The results with a *p*-value of <0.05 are considered statistically significant and annotated with “*”.

Please refer to Supplementary Tables S12–S14 for the selection process of effective mediators. Network MR analysis indicates that the class Negativicutes and order Selenomonadales may be associated with the onset of insomnia by potentially exacerbating pain symptoms, with a mediation proportion (95% CI) of 12.43% (0.47, 24.39%, Figure 3A). The genus *Oxalobacter* might contribute to the promotion of EDS by disrupting the adenine and adenosine salvage pathways, with a mediation proportion of 25.39% (1.84, 48.95%, Figure 3B). The genus *Allisonella* could increase the risk of OSA by potentially promoting obesity in patients, with a mediation proportion of 36.88% (17.23, 56.54%, Figure 3C), while the genus *Eubacterium xylanophilum* group may reduce the risk of OSA by possibly decreasing smoking behavior, with a mediation proportion of 7.70% (0.66, 14.74%, Figure 3D). The results of the sensitivity analysis indicate that the findings of the network MR are not influenced by weak instruments, horizontal pleiotropy, or reverse causation (Supplementary Tables S12–S16).

## 4 Discussion

In stage 1, we initially utilized UVMR to identify 14 types of gut microbiota that may influence five categories of sleep disorders. In stage 2, the results from an observational study confirmed significant differences in four of these microbiota between groups, aligning with the trends identified in UVMR analysis. In stage 3, the network MR analysis revealed that pain mediates the effect of the class Negativicutes and the order Selenomonadales in promoting insomnia. Additionally, disruptions in the adenine and adenosine salvage pathways mediate the role of the genus *Oxalobacter* in advancing EDS. Finally, obesity and smoking behavior were found to mediate the influence of the genus *Allisonella* and the genus *Eubacterium xylanophilum* group, respectively, on the incidence of OSA. Our research offers new insights and perspectives on novel pathways through which the gut microbiome regulates sleep disorders.

Previous studies have demonstrated that the gut microbiome influence sleep via the gut-brain axis, engaging intricate neural, immune, metabolic, and endocrine pathways (Wang et al., 2022). However, such studies have often been hindered by uncontrollable confounding factors, the possibility of bidirectional causation, and small sample sizes, leading to a lack of consistent findings and robust conclusions. MR offers a powerful solution to these challenges. Therefore, we firmly believe that MR is a compelling method for investigating the relationship between microbiomes and diseases.

Previous MR studies exploring the relationship between the gut microbiome and sleep have experienced several limitations: (1) they did not investigate the associations between the microbiome and less common sleep disorders, such as SWRD and iRBD. (2) The absence of non-MR findings as external validations failed to establish triangulation of evidence. (3) Previous research did not delve into how the microbiome may lead to sleep disorders. Our study, integrating MR analysis with observational research, effectively addresses these gaps. More importantly, by using the network MR analysis, we have opened new perspectives on how the gut microbiome influences sleep.

### 4.1 Class Negativicutes, inflammatory pain, and insomnia

Insomnia, the most prevalent sleep disorder, is characterized by difficulties in initiating or maintaining sleep (Buysse, 2013; Riemann et al., 2015). Pain is one of the most common risk factors for insomnia, as it can disrupt the equilibrium of the dopaminergic, serotonergic, and opioidergic systems, ultimately leading to sleep disturbances (Nijs et al., 2018). The Gram-negative class Negativicutes and its order Selenomonadales are notably capable of producing a biologically active component known as lipopolysaccharides (LPS), which is a primary inducer of inflammatory pain (Yin et al., 2020). In stage 1 of our study, UVMR analysis indicated that Negativicutes and Selenomonadales could promote insomnia, a finding supported by three secondary analysis methods and four supplementary analysis techniques, establishing “causality.” In stage 2, external validation through observational research revealed that despite lower alpha diversity in the insomnia group compared with healthy controls, the proportion of Negativicutes and Selenomonadales was significantly higher, bolstering the validity of our conclusions. In stage 3, the network MR analysis discovered that Negativicutes and Selenomonadales exacerbate insomnia by intensifying pain symptoms, with the LPS-induced inflammatory pain possibly identified as a critical mechanism leading to insomnia.

### 4.2 Genus *Oxalobacter*, adenosine salvage disruption, and EDS

EDS is characterized by an ongoing struggle to remain alert during the day (Pérez-Carbonell et al., 2022). EDS may be linked to an imbalance of neurochemical factors that govern sleep and alertness and accumulated sleep debt. Adenosine, a neurochemical that promotes sleep drive, accumulates between brain cells as wakefulness is prolonged, inhibiting the activity of brain regions associated with wakefulness and inducing sleep. Sleep duration extension allows the brain to convert adenosine back to ATP, reducing sleep drive and promoting alertness (Porkka-Heiskanen and Kalinchuk, 2011; Brown et al., 2012). This mechanism also explains why caffeine, as an adenosine receptor antagonist, can help maintain wakefulness.

For the first time, our research has identified that the gut microbiome may influence the occurrence of EDS by participating in the metabolism of adenosine. In stage 1, UVMR analysis pointed to the genus *Oxalobacter* as an independent risk factor for EDS. This conclusion was supported by 3 secondary analysis methods and 4 supplementary analysis techniques, establishing “causality”. In stage 2, our observational study could not effectively compare differences between groups due to the low abundance of the genus *Oxalobacter* in our subjects. In stage 3, through the network MR analysis, we discovered that the genus *Oxalobacter* may contribute to EDS by inhibiting the reuptake pathway of adenine and adenosine. The disruption of adenosine reuptake increases the concentration of adenosine in brain tissues, creating a prolonged sleep-promoting effect, which ultimately leads to the development of EDS.

Notably, *Oxalobacter* is Gram-negative, obligate anaerobic bacteria that specialize in degrading oxalate, a process crucial for maintaining oxalate homeostasis in the host. Low oxalate levels are considered a biomarker of sleep deprivation (Weljie et al., 2015). As an independent risk factor for EDS, increased levels of *Oxalobacter* seem to enhance oxalate degradation, thereby lowering oxalate levels in the body. This suggests that patients with EDS and sleep deprivation might exhibit reduced oxalate levels, presenting an apparent paradox. After searching on the Metorigin platform (Yu et al., 2022) and various other tools (Li et al., 2018, 2020; Lai et al., 2022), we identified that *Oxalobacter* is involved in the oxalate metabolic pathway R11617. This reversible reaction implies that *Oxalobacter* can promote not only oxalate degradation but also potentially oxalate synthesis under certain conditions. Whether *Oxalobacter* affects oxalate metabolism differently in the context of various sleep disorders remains to be clarified by future research.

### 4.3 Genus *Ruminiclostridium* 6, thermoregulation, and SWRD

Patients with SWRD typically experience difficulties initiating or maintaining sleep or challenges in waking up at appropriate times. The human sleep-wake cycle is governed by both the endogenous circadian system and sleep homeostasis. The maintenance of the circadian system relies on regulation by the suprachiasmatic nucleus, along with periodic variations of core body temperature and the release of wake-promoting signals (such as orexin) and sleep-promoting signals (such as cortisol and melatonin) (Meyer et al., 2022). The genus *Ruminiclostridium* has been implicated in the regulation of body temperature changes during sleep, thereby maintaining sleep rhythm (Thompson et al., 2021). Our study also indicates that *Ruminiclostridium* 6 may serve a protective function in preserving sleep rhythm in stage 1. This conclusion is bolstered by the findings from three secondary analysis methods and four supplementary analysis techniques.

### 4.4 Genus *Allisonella*, obesity, and OSA

OSA is characterized by repeated episodes of upper airway collapse, leading to complete or partial cessation of breathing. Before research on gut microbiota, OSA has largely focused on how OSA-induced intermittent hypoxia and sleep fragmentation disrupt the microbial balance, leading to various complications. Our study initiatively identifies gut microbiota as a potential precursor to OSA.

Obesity is a primary risk factor for OSA due to excessive fat deposition in the chest area, which increases respiratory resistance, compresses the airway, and causes airway narrowing. A significant finding from a large multicenter cohort study by Ecklu-Mensah et al. (2023) showed that obese patients exhibit notably higher levels of the genus *Allisonella*. Aranaz et al. (2021) reached similar conclusions in another observational study. In our research, in stage 1, we identified the genus *Allisonella* as an independent risk factor for OSA, a finding supported by three secondary analysis methods and four supplementary analysis techniques. In stage 2, we observed no significant differences in the prevalence of the genus *Allisonella* between OSA patients and HCs, likely due to the selection of participants with similar BMI to ensure comparable baseline information between groups. In stage 3, through network MR analysis, we discovered that the genus *Allisonella* indirectly increases OSA incidence by elevating patients’ BMI. The increase in the upper respiratory tract resistance due to fat deposition may play a significant role in this process. Additionally, previous MR studies have demonstrated that the genus *Allisonella* can also heighten the incidence of chronic obstructive pulmonary disease (COPD) (Wei et al., 2023), further substantiating our hypothesis.

### 4.5 Genus *Eubacterium xylanophilum* group, smoking behavior, and OSA

Our research also found that the genus *Eubacterium xylanophilum* group has a protective effect against OSA. Animal experiments and MR analyses suggest that the gut microbiota can affect reward- and stress-related behavior associated with tobacco use (Lee et al., 2018; Meckel and Kiraly, 2019; Fan et al., 2023). Meanwhile smoking, which can induce upper respiratory inflammation and cause relaxation of respiratory muscles, may trigger OSA (Lin et al., 2012). In our research, during stage 1, we discovered that the *Eubacterium xylanophilum* group acts as a protective factor against OSA, a conclusion supported by 3 secondary analysis methods and 4 supplementary analysis techniques, with the evidence level rated as “causality.” In stage 2, we also observed a significant decrease in the abundance of the *Eubacterium xylanophilum* group in OSA patients compared to HCs. In stage 3, network MR analysis revealed that this bacterial group indirectly reduces the incidence of OSA by decreasing smoking behavior in patients. This discovery significantly enhances our understanding, demonstrating that the microbiota can affect sleep not only through inflammatory, endocrine, and metabolic pathways of the gut-brain axis but also by influencing patient behavior.

### 4.6 Genus *Eubacterium coprostanoligenes* group, genus *Oscillibacter*, inflammation, and iRBD

IRBD is characterized by the lack of muscle atonia, disturbing dream content, and abnormal behaviors during REM sleep. Given that over 80% of iRBD cases evolve into Parkinson’s disease, multiple system atrophy, or dementia with Lewy bodies over a 16-year follow-up period (Schenck et al., 2013), iRBD is considered a precursor to alpha-synucleinopathies. Braak staging and previous studies indicate that the disruption of the intestinal barrier and local inflammatory responses can lead to the deposition of alpha-synuclein in the gut. This pathogenic alpha-synuclein may propagate along the enteric nervous system to the brain, ultimately leading to the occurrence of iRBD. These studies underscore the significant role of intestinal inflammation in the pathogenesis of iRBD.

The genus *Eubacterium coprostanoligenes* group and genus *Oscillibacter* have been found closely associated with immune-inflammatory responses (Guo et al., 2023; Gaudino et al., 2024). In an observational study focusing on HCs, first-degree relatives of iRBD patients, iRBD patients, and Parkinson’s disease patients with RBD symptoms, Yun Kwok Wing et al. observed a gradual increase in the abundance of the genus *Eubacterium coprostanoligenes* group and genus *Oscillibacter* with the progression of alpha-synucleinopathy. Similarly, Kinji Ohno et al. noted a significant rise in the abundance of these genera in iRBD patient groups compared to HCs in another observational study. In our research, during stage 1, UVMR analysis revealed that these genera could promote the onset of iRBD, a finding supported by three secondary analysis methods and various supplementary analysis techniques. In stage 2, compared to HCs, patients in the pRBD group exhibited significantly higher levels of the genus *Eubacterium coprostanoligenes* group, while increases in the genus *Oscillibacter* were observed but did not achieve statistical significance. Since stage 3 network MR analysis did not identify indirect causal pathways from these microbial groups to iRBD, further research is needed to elucidate the underlying mechanisms behind these associations.

### 4.7 Strengths and limitations

Compared to previous studies focusing on the gut microbiota and sleep disorders, our research offers several advantages: (1) robust conclusion: the superiority of the MR method allows us to effectively reduce confounding biases, reverse causation, and interference from insufficient sample sizes encountered in previous studies. Furthermore, we adopted stringent criteria for defining positive results, considering a conclusion to be preliminary only if supported by the IVW method and at least three out of four secondary analyses. Additionally, we used four novel models as supplementary analytical methods and conducted multiple sensitivity analyses to exclude results with heterogeneity, pleiotropy, and outliers. Most importantly, the results from observational studies and MR analyses together form triangulating evidence, significantly enhancing the credibility of our conclusions. (2) Rigorous mechanistic exploration: our study is the first to explore the indirect causal pathways through which the microbiome leads to sleep disorders, employing rigorous network MR analysis methods.

However, our study has several limitations across its three stages. In stage 1, (1) the stringent criteria for defining positive results might have led to overlooking some potential causal relationships. (2) All MR analysis data were derived from European populations, which limits the generalizability of our findings. Future MR analyses across different ethnicities are necessary to confirm whether the relationship between gut microbiota and sleep disorders is consistent. (3) While our study supports a link between gut microbiota and sleep disorders, it remains unclear whether these microbiota independently affect the onset of these disorders or interact with other microbiota, host genetic factors, and microenvironmental factors. Further research is needed to clarify these interactions. In stage 2, (1) we conducted an observational study with participants from Asian populations, while the UVMR results were primarily based on European populations, making racial differences an unavoidable factor. (2) The inclusion of participants did not account for the impact of lifestyle factors or work-related stress on sleep, potentially introducing confounding bias. (3) In the external validation, the selection of RBD patients was based on the RBD-HK questionnaire and was not confirmed through polysomnography (PSG). (4) This stage used a cross-sectional design and did not include patient follow-up. Monitoring these patients’ post-treatment to observe if symptom improvement correlates with changes in gut microbiota would provide stronger evidence for the UVMR results. (5) To enhance comparability between participant groups, we selected individuals with similar BMI levels. However, BMI could be a mediating factor in the effect of certain microbiota on sleep disorders, and adjusting for this variable might mask some true intergroup differences. In stage 3, further observational studies are needed to validate the differences in mediating factors, such as pain, smoking behavior, and plasma adenosine levels among patients with various sleep disorders.

## 5 Conclusion

In summary, the results from UVMR, observational study, and network MR analysis indicate that pain, obesity, adenosine salvage disruption, and smoking behavior mediate the effect of gut microbiota on sleep disorders.

## Data availability statement

The original contributions presented in the study are included in the article/Supplementary material, further inquiries can be directed to the corresponding author.

## Ethics statement

The studies involving humans were approved by the Ethics Committee of the Affiliated Hospital of Xuzhou Medical University. The studies were conducted in accordance with the local legislation and institutional requirements. The participants provided their written informed consent to participate in this study.

## Author contributions

F-JL: Conceptualization, Data curation, Formal analysis, Investigation, Methodology, Project administration, Resources, Software, Supervision, Validation, Visualization, Writing – original draft. R-YZ: Conceptualization, Data curation, Formal analysis, Investigation, Methodology, Project administration, Resources, Software, Supervision, Validation, Visualization, Writing – original draft. J-YL: Writing – review & editing. Y-NL: Writing – review & editing. Z-XZ: Writing – review & editing. LD: Writing – review & editing. Y-D-YL: Writing – review & editing. XL: Writing – review & editing. WZ: Writing – review & editing. G-YC: Writing – review & editing. C-YX: Supervision, Writing – review & editing.

## Footnotes

^1^ https://www.ukbiobank.ac.uk/

^2^ https://www.finngen.fi/fi

^3^ https://cloud.majorbio.com
